# Higher body weight-dependent neural activation during reward processing

**DOI:** 10.1007/s11682-023-00769-3

**Published:** 2023-04-04

**Authors:** Maike Richter, Sophia Widera, Franziska Malz, Janik Goltermann, Lavinia Steinmann, Anna Kraus, Verena Enneking, Susanne Meinert, Jonathan Repple, Ronny Redlich, Elisabeth J. Leehr, Dominik Grotegerd, Katharina Dohm, Harald Kugel, Jochen Bauer, Volker Arolt, Udo Dannlowski, Nils Opel

**Affiliations:** 1 Institute for Translational Psychiatry, University of Münster, Münster, Germany; 2 Institute for Translational Neuroscience, University of Münster, Münster, Germany; 3 Department of Psychology, Martin-Luther University of Halle, Halle, Germany; 4 University Clinic for Radiology, University of Münster, Münster, Germany; 5 Department of Psychiatry, University of Münster, Münster, Germany; 6 Department for Psychiatry, Psychosomatic Medicine and Psychotherapy, University Hospital Frankfurt, Goethe University, Frankfurt, Germany; 7 Department of Psychiatry, Jena University Hospital/Friedrich-Schiller-University Jena, Jena, Germany; 8 German Center for Mental Health (DZPG), Jena-Magdeburg-Halle, Germany; 9 Center for Intervention and Research on Adaptive and Maladaptive Brain Circuits Underlying Mental Health (C-I-R-C), Jena-Magdeburg-Halle, Germany

**Keywords:** fMRI, Insula, Reward processing, BMI, Obesity

## Abstract

Obesity is associated with alterations in brain structure and function, particularly in areas related to reward processing. Although brain structural investigations have demonstrated a continuous association between higher body weight and reduced gray matter in well-powered samples, functional neuroimaging studies have typically only contrasted individuals from the normal weight and obese body mass index (BMI) ranges with modest sample sizes. It remains unclear, whether the commonly found hyperresponsiveness of the reward circuit can (a) be replicated in well-powered studies and (b) be found as a function of higher body weight even below the threshold of clinical obesity. 383 adults across the weight spectrum underwent functional magnetic resonance imaging during a common card-guessing paradigm simulating monetary reward. Multiple regression was used to investigate the association of BMI and neural activation in the reward circuit. In addition, a one-way ANOVA model comparing three weight groups (normal weight, overweight, obese) was calculated. Higher BMI was associated with higher reward response in the bilateral insula. This association could no longer be found when participants with obesity were excluded from the analysis. The ANOVA revealed higher activation in obese vs. lean, but no difference between lean and overweight participants. The overactivation of reward-related brain areas in obesity is a consistent finding that can be replicated in large samples. In contrast to brain structural aberrations associated with higher body weight, the neurofunctional underpinnings of reward processing in the insula appear to be more pronounced in the higher body weight range.

**Supplementary Information:**

The online version contains supplementary material available at 10.1007/s11682-023-00769-3.

Obesity is a widespread cause of disability with severe public health implications (Mathers & Loncar, 2015). While diminished impulse control and altered valuation of rewarding food cues have been associated with dysfunctional eating behavior and obesity on a behavioral level (Maxwell et al., 2020), research also points toward aberrant neurobiological mechanisms underlying weight gain and obesity during reward processing (Donofry et al., 2020; García-García et al., 2014).

Overweight and obesity have repeatedly been linked to brain functional aberrations in response to reward cues (García-García et al., 2014; Opel et al., 2015). The insula, orbitofrontal cortex (OFC), and striatum have consistently been identified as key regions implicated in reward processing and obesity (Burger & Berner, 2014; Volkow et al., 2011; Wang et al., 2004). The striatum, including nucleus accumbens, caudate, and putamen, has been described as part of the “hedonic pathway” through its dopaminergic pathways associated with reward anticipation and function during general hedonic representation, particularly in network with the insula, anterior cingulate cortex and other midbrain structures such as the ventral tegmental area (VTA). The orbitofrontal cortex (OFC) is equally part of the hedonic pathway of overeating and obesity due to its role in decision-making and value appraisal of specific rewards such as palatable food (Kenny, 2011; Lee & Dixon, 2017). The insula, which is mostly known for its role in interoception and the homeostatic regulation of hunger and satiety (Carnell et al., 2012) has recently been identified as a region in which the expression of obesity susceptibility genes was most strongly enriched (Ndiaye et al., 2020). Moreover, the insular cortex also plays a crucial role in emotional processing as well as craving and feeding behavior, which makes this region especially interesting when examining reward processing in relation to body weight and obesity (Craig, 2009; Elliott et al., 2000; Yokum et al., 2011).

Previous investigations consistently find overactivation of reward-related regions in participants with obesity compared to participants within the normal weight range following rewards cues (Han et al., 2021; Stice & Burger, 2019; Yokum et al., 2011). Of note, these effects are not limited to food rewards but can equally be detected in response to non-food rewarding stimuli (Opel et al., 2015). However, it remains unclear, whether an overactivation of the reward circuit in response to reward cues can be found throughout the BMI range or if it is particular to clinical obesity. Evidence from brain structural investigations consistently reveals continuous body weight related gray matter atrophy and cortical thinning (Opel et al., 2017; Raji et al., 2010; Shaw et al., 2018). This association has been linked to a number of potential mechanisms associated with elevated body weight, such as fitness level, cardiac function or inflammation (Bobb et al., 2014; Hayes et al., 2014; Jefferson, 2010), which suggests potential atrophic effects of increased adiposity on brain structure. This is in line with the food addiction model of overeating and obesity which describes these common findings as potential effects of a high-fat/sugar diet, the overconsumption of food, and the adipose state (Smith & Robbins, 2013). However, the model also describes a cognitive component as a potential mechanism for the development and maintenance of obesity, related to motivation, response to rewarding cues and eating behavior. These cognitive processes may be particularly aberrant in the highest weight range, where overeating and potential food addiction may be most pronounced (Smith & Robbins, 2013) and therefore especially relevant when investigating brain functional aberrations related to adiposity.

Most previous research on the relationship between body weight and neural reward processing has investigated small sample sizes (García-García et al., 2014; Han et al., 2021) and typically either compared individuals with obesity with a normal weight control group (Han et al., 2021; Opel et al., 2015) or made no distinction between overweight and obese weight groups (García-García et al., 2014; Meng et al., 2020). Studies that consider the entire BMI range in larger samples do not typically investigate whether the effects occur as a function of higher body weight, or whether they are driven by a particular weight group (Beyer et al., 2021; Bhutani et al., 2021). Nevertheless, a non-linear relationship between reward sensitivity and body weight has long been discussed, though the direction of effects has not been uniform across investigations, with some authors describing blunted reward response in individuals with obesity (Davis & Fox, 2008; Horstmann et al., 2015) – hypothesizing that individuals overeat to compensate for the reduced neural reward response. Another investigation in adolescents with overweight and obesity found no association between continuous BMI and neural response to high vs. low calorie drinks but instead revealed overactivation in reward-related areas in association with insulin resistance (Feldstein Ewing et al., 2017). Insulin resistance as a common comorbidity of the obese state (Ye, 2013) may therefore also play a role in setting reward processing in individuals with obesity apart from overweight and normal weight. Verdejo-Román et al. (2017) found impaired reward learning during reward anticipation in participants with obesity compared to overweight and normal weight groups and a subsequent overactivation of striatal areas after reward receipt, which the authors attribute to severity related neuroadaptations, further underlining the unique role clinical obesity might have during reward processing. This is in line with food addiction models which posit enhanced reward circuitry response along with impaired executive control circuitry function and decreased inhibitory control over eating behavior in response to highly palatable foods as the neural basis for developing obesity (Smith & Robbins, 2013; Volkow et al., 2013).

Although sparse, this evidence in concert with theoretical models of food addiction lends support to the hypothesis that, in contrast to the linear association between body weight and brain structural aberrations, previously established effects regarding an overactive reward circuit may be particularly pronounced in higher body weight ranges or even specific for clinical obesity, potentially due to secondary effects of the adipose state such as aberrant hormone levels, gut microbiome, but also due to cognitive processes regarding reward and feeding behavior (Devoto et al., 2018; Dong et al., 2022; Murray et al., 2014; Reinehr et al., 2008; Stice & Burger, 2019). Our own group was previously able to show overactivation of the OFC, insula and putamen when contrasting participants with obesity with a normal weight control group in a sample of 29 participants per group (Opel et al., 2015). It is warranted to attempt replication of this study with a bigger sample size, while moving away from merely contrasting participants with normal weight and obesity. Although BMI cut-offs are well established in clinical practice to categorize and quantify health risk (Weir & Jan, 2019), and previous research has relied on these categories to group participants, the established effects regarding enhanced reward response in obesity should also be investigated without predefined cut-offs. In this work, we therefore decided to conduct a number of analyses to test continuous BMI effects, weight class effects as well as a supplemental analysis to investigate a data-driven weight cut-off associated with stronger reward activation.

We hypothesized that

1) BMI as a continuous measure would be positively related to activation of reward-related areas (OFC, insula, VTA, striatum) during reward processing.

2) In line with the aforementioned evidence for obesity-specific reward processing alterations, we further hypothesized that this effect would mainly be driven by the group of participants with a BMI in the obese range.

3) Moreover, we expected the association between BMI and reward circuitry activation to follow a non-linear trend, with more pronounced activation in the highest BMI range.

## Methods

### Participants

Participants were recruited at the Department of Psychiatry, University of Münster, Germany as part of the Münster Neuroimaging Cohort from October 16, 2009 – May 19, 2017. The original subsample comprised 412 participants, 26 of which were excluded due to missing height and weight. Due to excessive head movement, 3 further subjects were excluded (exclusion criterion > 3 mm/3°), leaving a final sample of 383 participants (female *n* = 189; *M*_Age_ = 39.21; *M*_BMI_ = 24.64; see Table 1). All participants were free of mental disorders, which was verified with the Structured Clinical Interview for DSM-IV (Wittchen et al., 1997). Any history of neurological (e.g., concussion, stroke, tumor, neuroinflammatory diseases) and medical (e.g., cancer, chronic inflammatory or autoimmune diseases, heart diseases, diabetes mellitus, infections) conditions as well as regular medication intake were exclusion criteria. BMI was calculated from self-reported height and weight.

**Table 1:** Sociodemographic and clinical characteristics of the whole sample and subsamples according to weight group. Number of available data, means, SD and range

			*N*	*Mean (SD)*	*SD*	*Range*
**Age**		**Whole sample**	**383**	**39.2 (11.3)**	**19–59**	**39.2 (11.3)**
		*Normal weight*	236	36.9 (11.8)	20–59	36.9 (11.8)
		*overweight*	113	43.2 (9.5)	21–59	43.2 (9.5)
		*obese*	34	41.9 (9.6)	19–56	41.9 (9.6)
**Sex (m/f)**		**Whole sample**	**194/189**			
		*Normal weight*	110/126			
		*overweight*	67/46			
		*obese*	17/17			
**BMI**		**Whole sample**	**383**	**24.6 (4.0)**	**18.2–42.2**	**24.6 (4.0)**
		*Normal weight*	236	22.1 (1.7)	18.2–24.9	22.1 (1.7)
		*overweight*	113	27.2 (1.5)	25.1–29.9	27.2 (1.5)
		*obese*	34	33.5 (2.9)	30.0–42.2	33.5 (2.9)

*Note.* SD, standard deviation.

### Stimulus materials

A modified version of a commonly used card guessing paradigm (Redlich et al., 2015) was used to detect brain activity related to reward processing. A detailed description of the pseudorandom block-design paradigm can be found in the supplementary material.

### fMRI data acquisition and analysis

T2* functional data were acquired using a 3 Tesla scanner (Gyroscan Intera 3T, Philips Medical Systems, Best, NL), using a single-shot echoplanar sequence, with parameters selected to minimize distortion in the region of central interest, while retaining adequate signal-to-noise ratio and T2* sensitivity. Pre-processing of our functional data included realignment, unwarping, and spatial normalization to MNI-space as well as smoothing with a Gaussian kernel of 6 mm full-width at half-maximum as described in our previous work (Opel et al., 2015). To isolate neural response during the different blocks (control, win, lose), onsets and durations of the corresponding experimental conditions were modelled using a canonical hemodynamic response function. This was done in the context of the general linear model including corrections for serial correlations and application of a high-pass filter of 128 s to remove low-frequency noise. For each subject, first-level analyses were conducted yielding a contrast-image for the “win > control” condition. More details on fMRI the data acquisition can be found in the supplementary material. Stimulus materials, fMRI procedure, preprocessing protocols, and first-level analyses remained unchanged from the procedures used by Opel et al. (2015), in order to ensure comparability for this replication attempt in a larger sample size.

### Second-level analyses

The OFC, bilateral insula, nucleus accumbens, caudate, putamen and the VTA were combined as one single region-of-interest (ROI) mask, in all analyses from steps 1) to 3). The mask was created with the Wake Forest University PickAtlas (Maldjian et al., 2003) using the AAL-atlas definitions (Tzourio-Mazoyer et al., 2002) with the bilateral labels: anterior, middle, posterior, and medial orbital frontal gyrus, insula, caudate, putamen, VTA. A statistical threshold of p < .05, with voxel-level family wise error (FWE) correction, was used in the following analyses.

1. To address our hypothesis of BMI-associated altered reward processing, we performed a regression analysis, with BMI as covariate of interest and age and sex as nuisance regressors.
2. Analysis 1) was repeated with the same covariates, including only participants from the normal weight and overweight BMI range.
3. To investigate whether potential BMI effects were driven by a particular weight class, a one-way ANOVA was conducted, including three groups according to WHO definition (normal weight: ≥ 18, < 25 kg/ m2; overweight: ≥ 25, < 30 kg/m2; obese: ≥ 30 kg/m2) (Weir & Jan, 2019) with age and sex as covariates.
4. Additional exploratory whole brain analyses for all models were performed to identify potential effects outside of the reward circuit with an uncorrected threshold of p < .001.
5. In order to investigate the association between BMI and insula activation further, an additional segmented regression analysis was performed, entering BMI as predictor, the extracted BOLD from the insula peak voxel as dependent variable and BMI = 30 as a suggested cut-off point (see supplementary material for more detail regarding segmented regression).
6. Additional supplementary analyses were conducted to investigate childhood maltreatment and novelty seeking, two potential confounders commonly associated with BMI (see supplementary material for more detail).

## Results

**Fig. 1:**
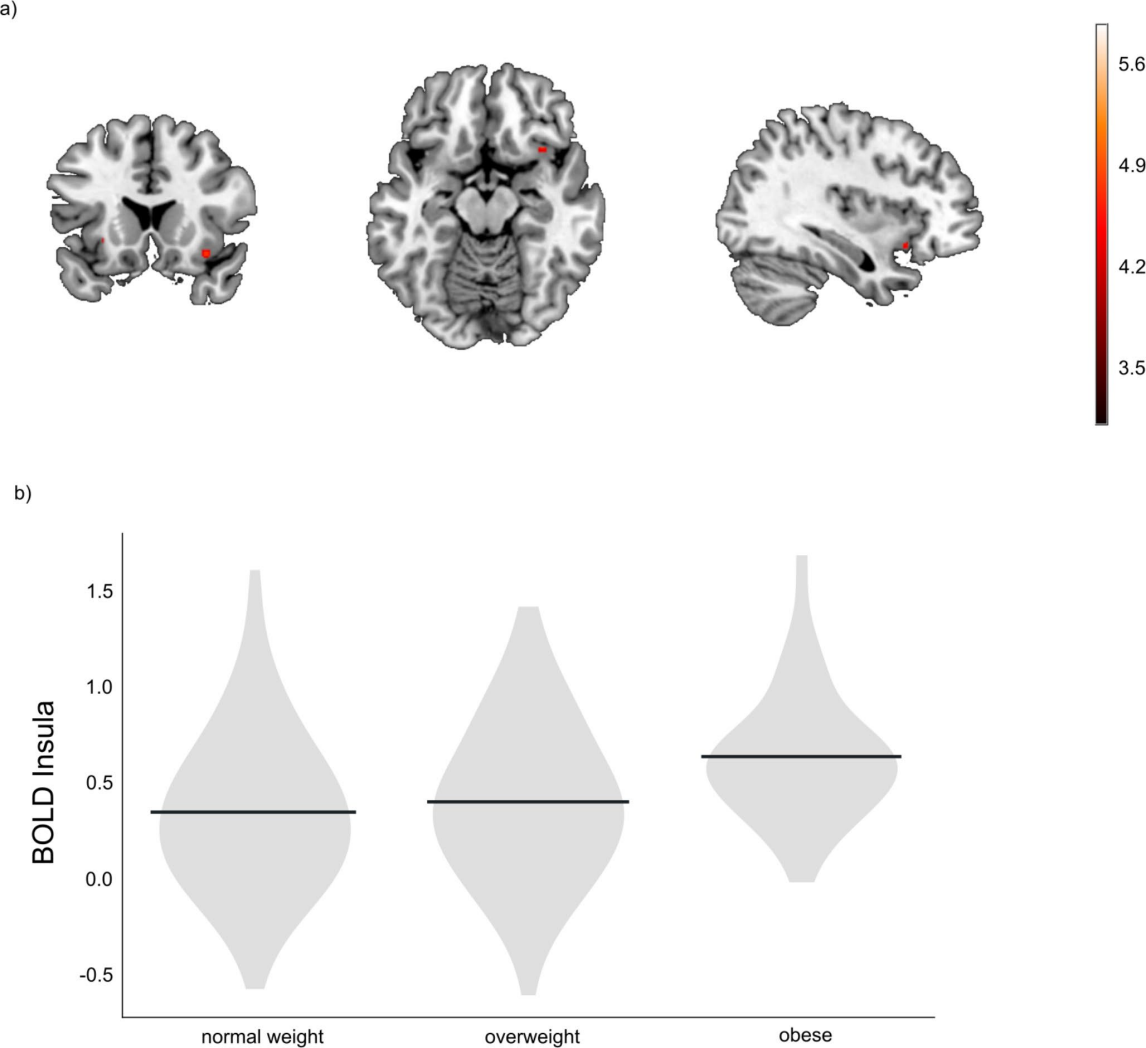
**(a)** Positive effect of BMI on neural responsiveness to reward in the insula. Results from the region-of-interest-analysis of the one-way ANOVA for the obesity > normal weight contrast are shown at MNI coordinates x = 36, y = 18, z = -14. Family-wise error corrected results at a voxel threshold of p < .05 are presented. Color bar: t-value. **(b)** Violin plot depicting the density and group means of extracted insula BOLD values (3 mm around the peak voxel) for each weight group from the ANOVA model

1. The multiple regression analysis revealed a significant positive effect of BMI in the right (x = 36, y = 18, z = -14; *t*(379) = 4.66; *k* = 9; *p*_FWE_ = 0.007) and left (x = -28, y = 18, z = -4; *t*(379) = 4.30; *k* = 3; *p*_FWE_ = 0.029) insula.
2. When excluding the participants with obesity from analysis 1), no significant positive effects of BMI on reward response could be found.
3. When examining the weight groups separately with a one-way ANOVA, there was a main effect of group in the insula (x = 34, y = 18, z = -14; *F*(2, 378) = 11.42; *k* = 1; *p*_FWE_ = 0.04). Participants with obesity exhibited higher activation in the right (x = 34, y = 18, z = -14; *t*(378) = 4.78; *k* = 10; *p*_FWE_ = 0.005) and left (x = -28, y = 20, z = -4; *t*(378) = 4.29; *k* = 6; *p*_FWE_ = 0.033) insula compared to the normal weight group (contrast obese > normal weight, see Fig. 1). The group with obesity also showed higher activation in the right caudate (x = 12, y = 22, z = 2; *t*(378) = 4.27; *k* = 2; *p*_FWE_ = 0.029) compared to the overweight group (contrast obese > overweight). There were no significant differences in activation between the overweight group compared to lean participants (contrast overweight > normal weight) in our regions of interest either at an uncorrected voxel-threshold or after FWE-correction.
4. The exploratory whole-brain regression analysis revealed further positive associations between BMI and higher neural activation in a cluster comprising the precuneus (Table 2). Similarly, the exploratory whole-brain ANOVA revealed additional clusters with higher activation (obese > normal weight; obese > overweight) in prefrontal, orbitofrontal, and striatal areas (Table 3). These effects were solely found at an uncorrected voxel-threshold of *p* < .001, and no significant clusters remained after FWE-correction.
5. The results from the segmented regression analysis remained inconclusive as the model did not reach significance (see supplementary material).
6. Childhood maltreatment and novelty seeking did not meaningfully confound results as is detailed in the supplementary material.

**Table 2:** Exploratory whole-brain results of the regression analysis (positive effect of BMI) including age and sex as covariates

	*k*	*MNI (at peak)*			*Side*	T*-value*
		*x*	*y*	*z*		
Insula	56	36	18	-14	R	4.66
Insula	32	-28	18	-4	L	4.30
Precuneus	33	4	-60	24	R	3.91

*Note.* All reported whole-brain analyses with voxel-threshold p < .001 and minimum cluster volume threshold k ≥ 30. Coordinates based on MNI atlas. Abbreviations: BMI, body mass index; k, cluster size; L, left; MNI, Montreal Neurologic Institute; R, right.

**Table 3:** Overview of exploratory whole-brain analyses of the one-way ANOVE including age and sex as covariates

	*k*	*MNI (at peak)*			*Side*	T*-value*
		*x*	*y*	*z*		
**Exploratory whole-brain results of the one-way ANOVA (obese > normal weight)**						
Insula/ Inferior frontal gyrus, orbital part	127	34	18	-14	R	4.78
Superior frontal gyrus	91	20	58	-4	R	4.36
Insula	62	-28	20	-4	L	4.29
Middle frontal gyrus / Superior frontal gyrus	103	-26	48	6	L	4.21
Caudate	34	-12	26	12	L	3.75
Inferior frontal gyrus, triangular	39	-38	32	22	L	3.46
**Exploratory whole-brain results of the one-way ANOVA (obese > overweight)**						
Caudate	35	12	22	2	R	4.32
Superior frontal gyrus	74	18	58	-2	R	4.20
Insula	84	34	18	-14	R	3.93
Middle frontal gyrus	86	-26	46	8	L	3.63
Caudate	30	-12	22	6	L	3.54
**Exploratory whole-brain results of the one-way ANOVA (overweight > normal weight)**						
Middle temporal pole	13	34	14	-32	R	4.04
Parahippocampal gyrus / Fusiform gyrus	10	-24	-8	-32	L	3.69
Superior frontal gyrus	12	-16	26	50	L	3.58

*Note.* All reported whole-brain analyses with voxel-threshold p < .001 and minimum cluster volume threshold k ≥ 30, minimum cluster volume for the overweight > normal weight contrast k ≥ 10. Coordinates based on MNI atlas. Abbreviations: BMI, body mass index; k, cluster size; L, left; MNI, Montreal Neurologic Institute; R, right.

## Discussion

With this study, we aimed to shed light on the hitherto unanswered question whether brain functional responses to reward are associated with body weight over the entire weight spectrum or if reward circuit overactivation is more pronounced in clinical obesity.

We were able to replicate previous findings on the association of BMI and hyper-responsiveness of the insula to rewarding stimuli in a large sample of more than 380 participants (Han et al., 2021; Opel et al., 2015). Moreover, this effect appeared to be driven by participants in the higher BMI range. Our results complement those by Verdejo-Román et al. (2017) who found an obesity-specific overactivation in striatal areas in response to food rewards. Individuals with obesity also displayed greater activation of the rostral-ventral pons and nucleus accumbens after monetary reward feedback compared to overweight and normal weight groups in their investigation.

While most other previous studies did not investigate both continuous and weight group specific effects in fMRI, a non-linear relationship between body weight and other measures of neural reward has been found and discussed previously, especially in the context of dopamine receptor binding potential: Morbid obesity has been linked to less striatal dopamine (D2) receptor availability when compared to participants with a lean BMI and a negative association between D2 and BMI was only significant in the group with obesity in another investigation (Wang et al., 2001). More recent studies also argue against a linear relationship between body weight and D2 receptor availability and instead suggest differential D2 binding potentials and differing levels of reward sensitivity dependent on the degree of obesity (Cosgrove et al., 2015; Horstmann et al., 2015). Although the direction of effects in obesity was not the same across these studies and evidence for an inversed U-shaped association between body weight and dopamine availability was found, this nevertheless lends support to the theory of non-linear weight class dependent effects in reward processing.

Aside from dopamine, reward-related neural activation alterations in clinically relevant obesity could be associated with peripheral hormone levels (particularly leptin, ghrelin and insulin) which are disturbed in obesity (Leigh & Morris, 2018) and play a role in homeostasis, feeding behavior and reward processing: The ventral striatum might become desensitized to leptin’s inhibitory effect after long-term dietary changes and with increased adiposity (Jastreboff et al., 2014). Leptin has also been associated with a higher reward response in the insula in participants with obesity compared to lean participants (Jastreboff et al., 2014). The ghrelin system, which is related to appetite control and metabolic regulation as well as reward response, has been found to be impaired in obesity (Reinehr et al., 2008). Low ghrelin levels in subjects with obesity predicted hyperactivity in the reward circuit compared to lean individuals in a recent investigation (Bogdanov et al., 2020).

However, some evidence also lends support to the theory of continuous body weight-dependent reward system aberrations. Reinehr and colleagues (2008) found low ghrelin levels in obesity to not increase after weight loss. This might signify a lasting consequence of the previous adipose state and therefore constitute a risk factor for not maintaining weight loss, but it could also be evidence against the obesity-specificity of low ghrelin. Another investigation revealed that obesity-prone individuals fail to attenuate insula hyperactivity after ingesting a meal compared to their obesity-resistant counterparts, thus indicating neural overactivation of the insula as a potential risk factor for obesity that may be present in non-obese individuals (Cornier et al., 2013). A recent meta-analysis even revealed no differences between participants on the normal weight and the obese weight spectrum in reward processing and only reported age-related effects (Morys et al., 2020). Similarly inconsistent results emerge when examining other measures of neuronal function, such as functional connectivity (Beyer et al., 2021; Geha et al., 2017).

Some limitations should be considered when interpreting the results of this study. Due to exclusion criteria, participants were free of eating disorders and somatic disorders commonly associated with adiposity, such as hypertension and diabetes. Although this allows the observation of unconfounded effects of adiposity, it does not reflect the clinical reality and our sample may even represent a particularly resilient subgroup of participants with obesity. However, brain structural aberrations associated with cardiovascular risk factors such as hypertension and hyperlipidemia can be found in asymptomatic patients, suggesting that the absence of cardiovascular symptoms may not necessarily be associated with brain health (Friedman et al., 2014). Nevertheless, investigations on less homogeneous groups are needed when clinical applications of reward processing in obesity are researched in the future. Moreover, individuals with morbid obesity were difficult to recruit due to scanner weight and circumference restrictions – an increasingly common problem in radiology (Carucci, 2013) – leading to a systematic exclusion of participants at the highest BMI range with more severe weight complications. The sample with obesity in our study was therefore significantly smaller than that of the lean and overweight groups and our results should be interpreted with caution, especially in the context of whether our findings are specific for clinical obesity, which cannot be answered definitively.

Another limitation is the use of self-reported height and weight which can be biased particularly in more extreme body weight ranges (Maukonen et al., 2018). Previous analyses from a large-scale neuroimaging cohort revealed only marginal differences in effect sizes of brain structural associations with BMI when correcting for self-report bias and comparing results with a different sample in which height and weight were directly measured (Opel et al., 2017). Although we analyzed brain functional alterations in this study, this may suggest that the use of self-reported data for BMI measures did not skew our results significantly. Evidence suggests that health risk estimates remain virtually unchanged whether they are based on self-reported or measured BMI (Stommel & Schoenborn, 2009) and a recent analysis of BMI self-report bias in large cohort studies such as the one this sample was derived from also concluded that self-reported BMI is a valid measure across genders and socio-demographic groups (Hodge et al., 2020). Nevertheless, critical assessment regarding the use of self-reported height and weight remains crucial when interpreting our results, particularly due to larger bias in obese weight groups.

Although our sample size of n = 383 was large compared to the median neuroimaging sample size of n = 25, a recent publication suggests that reproducible effects in this field require thousands of participants (Marek et al., 2022). The problem of inflated effect sizes and lack of reproducibility at small sample sizes is considerable and we encourage caution when interpreting our results, especially in light of the smaller number of individuals with obesity. However, it should be pointed out that this study in itself serves as a replication attempt of a previous investigation in a smaller sample (Opel et al., 2015). In light of the replication crisis in the field, this lends support to the robust nature of our results.

As a methodological limitation it should be pointed out that the preprocessing protocol used in this study has remained unchanged since the beginning of this long-running cohort study in order to ensure comparability between participants. More recent investigations make use of protocols that allow for a greater degree of motion control (Maknojia et al., 2019). Although we adhered to an established protocol that was used in previous publications (Redlich et al., 2015; Zaremba et al., 2018) and provides good control for head motion, this limitation should be considered when interpreting our results.

Due to the cross-sectional nature of our investigation, no assumptions about causality can be made. It remains unclear whether alterations in reward processing among individuals with obesity are a risk factor for the development of adiposity or its sequelae. The food addiction model posits two components involved in the development and maintenance of obesity: consequences of the overconsumption of high-fat/high-sugar foods as well as somatic factors such as inflammation associated with neural atrophy on the one hand, and cognitive processes related to reward cues and reward learning incentivizing the overconsumption of food on the other hand (Smith & Robbins, 2013). Longitudinal evidence has shown that higher activity in reward regions predicts future weight gain and is associated with poorer response to weight-loss interventions, which suggests that aberrant reward processing may constitute a vulnerability for the development of obesity (Lin & Qu, 2020; Stice & Burger, 2019). Moreover, investigations on familial predisposition and genetic risk for obesity revealed that higher neural response to food cues could be detected in non-obese individuals at risk for obesity (Carnell et al., 2017; Kühn et al., 2016). However, other longitudinal investigations have also revealed obesity-specific resting state dysfunction in the OFC to recover after bariatric surgery (Li et al., 2018). After such a surgery, functional connectivity between regions related to cognitive control over food and bodily perception was reshaped and participants showed a reduction in reward-driven behavior (Olivo et al., 2017), suggesting these aberrations in reward processing to be byproducts of the obese state. Reward-based eating drive was associated with BMI, predicted earlier obesity onset and more weight gain as well as more frequent weight fluctuations in another investigation, although no neural measures were available (Epel et al., 2014). More evidence on the longitudinal association of obesity and reward-related neural activation is needed. In addition to longitudinal studies investigating weight changes over time, future research should focus on sampling broader age ranges. Adolescence has been described as a period of heightened reward sensitivity during which dopaminergic innervation from the VTA to the prefrontal cortex has been found to mature, and the foundation for future weight gain and obesity may be built (Lowe et al., 2020). A recent longitudinal investigation in children found a negative association between BMI and gray matter in regions involved in reward evaluation over a period of 2 years. The authors hypothesize that structural changes in the PFC may lead to ensuing impairments in self-regulation that exacerbate weight gain, particularly pointing out the potential detrimental effects of remaining in the obese weight range for a long time (Jiang et al., 2022).

It is therefore of great interest to investigate reward processing and body weight from childhood to adulthood to gain a clearer understanding of directionality of effects and mechanisms of action.

Considering the clinical application of these findings, it may be worthwhile to take reward-related behavior into account when targeting obesity and overweight. Neural aberrations during reward processing in individuals with clinical obesity are likely to have behavioral consequences. Reward-based weight-loss interventions should therefore be considered when targeting patient these patient groups (Mason et al., 2016; Mata et al., 2017).

In conclusion, this study offers important new insights into the association between neural reward processing and body weight that could inform future longitudinal research and the development of targeted reward-related interventions to tackle the ongoing obesity epidemic.

## Electronic supplementary material

Below is the link to the electronic supplementary material.

Supplementary Material 1

## Data Availability

Not applicable.
