# Supplementary material for: Higher body weight-dependent neural activation during reward processing
Source: Brain Imaging Behav. 2023 Apr 4;17(4):414–24. doi: 10.1007/s11682-023-00769-3 (PMC10435630; doi:10.1007/s11682-023-00769-3)
Supplement: Supplementary file 1 — Supplementary Material 1 [file 11682_2023_769_MOESM1_ESM.docx]

# Supplementary material

# Supplementary methods

## fMRI Data Acquisition

Volumes consisting of 34 slices were acquired, in plane matrix 64 x 64, resulting in cubic voxels of 3.6 mm edge length, repetition time = 2.1 s, echo time = 30 ms, flip angle = 90°). The slices were tilted 25° from the anterior commissures/posterior commissures line to minimize drop out artefacts in the mediotemporal and orbitofrontal region. The paradigm presentation was initially projected to the rear-end of the scanner (Sharp XG-PC10XE with additional high frequency shielding) for the first 25 participants that were measured in this sample. Starting in January 2012, the paradigm presentation was projected to the rear-end of the scanner from outside the Faraday cage (JVC DLA-HD1), due to modernization of the setup. Visibility of the paradigm stimuli was unaffected by this change. During the experiment, subjects lay supine in the MRI scanner with the response box in their right hand. The head position was stabilized with a vacuum head cushion. Data were analyzed using statistical parametric mapping software (SPM12, Wellcome Centre for Human NeuroImaging (WCHN), in the Institute of Neurology at University College London (UCL), UK; <http://www.fil.ion.ucl.ac.uk/spm>).

## Paradigm

To measure reward outcome feedback during reward processing, a card-guessing paradigm (Forbes et al. 2009; Opel et al. 2015; Redlich et al. 2015) was used as described by Redlich et al. (2015). Participants were instructed that their guessing performance would primarily affect monetary reward while reaction times could be neglected, although the actual outcome was set at 10€.

The pseudorandom block design included 9 blocks, each comprising 5 trials: 3 "win" blocks (block 1, 4, 7), 3 "lose" blocks (block 2, 5, 8) and 3 control blocks (block 3, 6, 9). Participants were given 3 seconds within each trial to decide whether the presented card had a value lower or higher than 5 by pressing one of two buttons on the response box in their hand with either their index (higher) or middle (lower) finger. Subsequently, subjects were able to see the numerical value of the card as well as feedback (green upward-oriented arrow for positive feedback, red downward-oriented arrow for negative feedback), for 0.5 seconds respectively. By pressing either button on the response box, subjects had to then validate positive feedback only. Whenever odd-numbered stimuli were presented, a crosshair followed and was shown for 1.5 seconds, while it was presented for 2.5 seconds subsequent to even-numbered stimuli, leading to total trial duration times of 5.5 seconds and 6.5 seconds.

In between “win” and “lose” blocks, control blocks were shown, in which subjects were instructed to respond via button pressing whenever an ‘x’ (3 seconds) occurred. Next, an asterisk (0.5 seconds), a yellow circle (0.5 seconds) and a crosshair were presented (likewise 1.5 seconds for odd-numbered stimuli and 2.5 seconds for even-numbered stimuli). At the beginning of each block, subjects were given short instructions (3 seconds), so that block length reached a total of 32.5 seconds for odd-numbered blocks and 33.5 seconds for even-numbered blocks, leading to a total task length of 296.5s

Overall, positive feedback dominated the three “win” blocks (four trials, 80% correct), while negative feedback prevailed all three “lose” blocks (four trials, 80% false). Subjects were instructed that positive feedback would result in an additional, fictional amount of 1€, whereas negative feedback would lead to a subtraction of 50 cents.

## Analyses

1. In order to investigate the association between BMI and insula activation further, the eigenvalues of insula blood-oxygen-level dependent (BOLD) signal from the main regression analysis were extracted using the eigenvariate function in SPM with a 3 mm sphere around the peak voxel. A segmented regression analysis was performed with the ‘segmented’ package in RStudio (Muggeo 2008) entering continuous BMI as predictor and peak voxel BOLD as dependent variable. BMI = 30 was selected a priori as a breakpoint to investigate whether the regression slope of insula reward response differed in obese individuals but a data-driven breakpoint was also estimated. Segmented regression is a method in which the independent variable is partitioned into intervals and a separate regression line is fitted to each partition. It is useful when the independent variable is clustered into different groups that may display different relationships with the dependent variable or if an abrupt change in the relationship is to be expected (Muggeo 2003) and was therefore chosen to investigate the expected change in the relationship between BMI and reward activation in the highest BMI range.

# Supplementary results

1. The segmented regression analysis revealed an estimated break point at BMI = 26, *SE* = 5.04 (see Figure S1), although the change in slope at this break point did not reach significance (Adjusted R² = 0.038, t(379) = 1.22, p = .22).

**Figure S1**

Results of the segmented regression analysis with an estimated breakpoint of BMI = 26


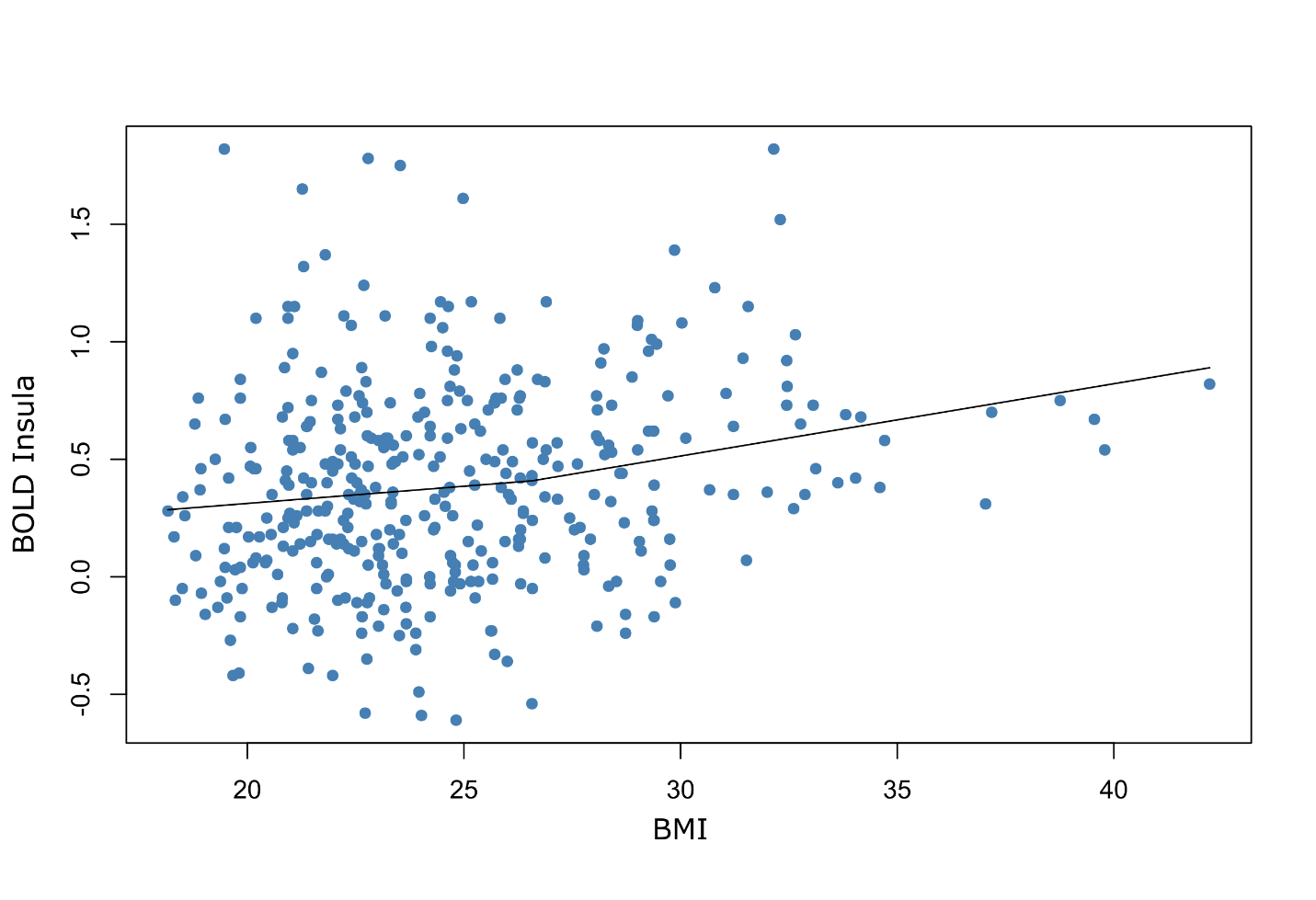


**Figure S2**


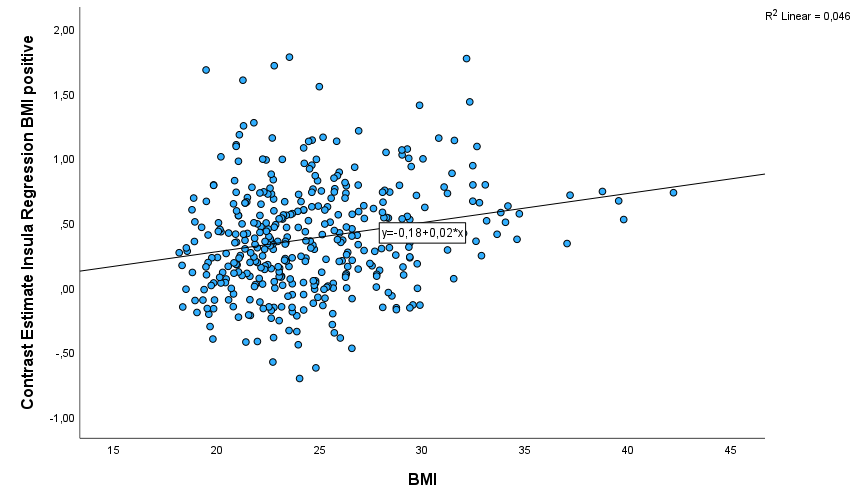
BMI and contrast estimates for each subject averaged over each voxel in the insula’s significant cluster from the regression analysis (positive effect of BMI contrast)

**Figure S3**

**
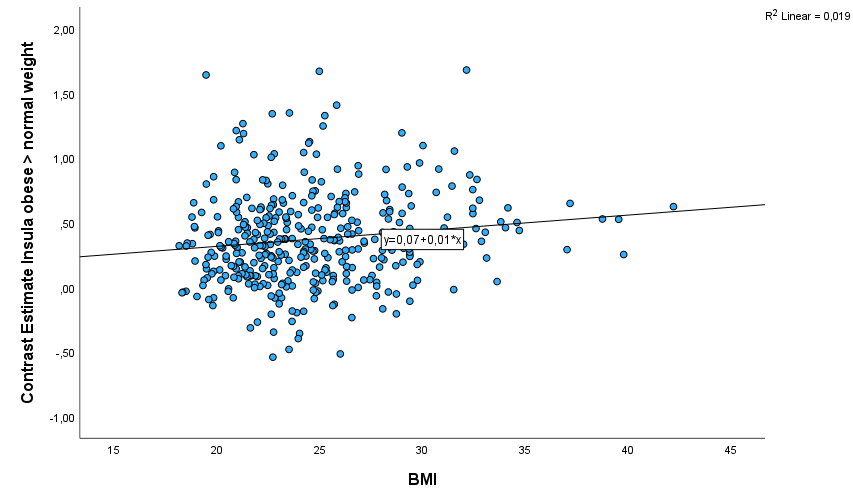
**BMI and contrast estimates for each subject averaged over each voxel in the insula’s significant cluster from the ANOVA (obese > normal weight contrast)

# References

Forbes, E. E., Brown, S. M., Kimak, M., Ferrell, R. E., Manuck, S. B., & Hariri, A. R. (2009). Genetic variation in components of dopamine neurotransmission impacts ventral striatal reactivity associated with impulsivity. *Molecular Psychiatry*, *14*(1), 60–70. https://doi.org/10.1038/sj.mp.4002086

Muggeo, V. M. R. (2008). Segmented: an R package to fit regression models with broken-line relationships. *R news*, *8*(1), 20–25.

Opel, N., Redlich, R., Grotegerd, D., Dohm, K., Haupenthal, C., Heindel, W., et al. (2015). Enhanced neural responsiveness to reward associated with obesity in the absence of food-related stimuli. *Human Brain Mapping*, *36*(6), 2330–2337. https://doi.org/10.1002/hbm.22773

Redlich, R., Dohm, K., Grotegerd, D., Opel, N., Zwitserlood, P., Heindel, W., et al. (2015). Reward Processing in Unipolar and Bipolar Depression: A Functional MRI Study. *Neuropsychopharmacology*, *40*(11), 2623–2631. https://doi.org/10.1038/npp.2015.110
